# Supplementary material for: Color construction of multi-colored carbon fibers using glucose
Source: Nat Commun. 2024 Mar 4;15:1979. doi: 10.1038/s41467-024-46395-5 (PMC10912437; doi:10.1038/s41467-024-46395-5)
Supplement: Supplementary file 1 — Supplementary Information [file 41467_2024_46395_MOESM1_ESM.pdf]

Supplementary information

Color construction of multi-colored carbon fibers using glucose

Sijie Zhou[1, 2], Chunhua Zhang[1], Zhuan Fu[1, 3], Qimeng Zhu[1], Zhaozixuan Zhou[1], Junyao Gong[1], Na Zhu[1], Xiaofeng Wang[1], Xinjie Wei[1], Liangjun Xia[1] * & Weilin Xu[1] *

[1] State Key Laboratory of New Textile Materials and Advanced Processing Technologies, Wuhan Textile University, Wuhan 430200, China

[2] College of Textiles, Donghua University, Shanghai 201620, China

[3] College of Textile Science and Engineering, Zhejiang Sci-Tech University, Hangzhou 310018, China

*Corresponding authors: State Key Laboratory of New Textile Materials and Advanced Processing Technologies, Wuhan Textile University, Wuhan 430200, China
E-mail addresses: liangjun_xia@wtu.edu.cn (L. Xia); weilin_xu@wtu.edu.cn (W. Xu)

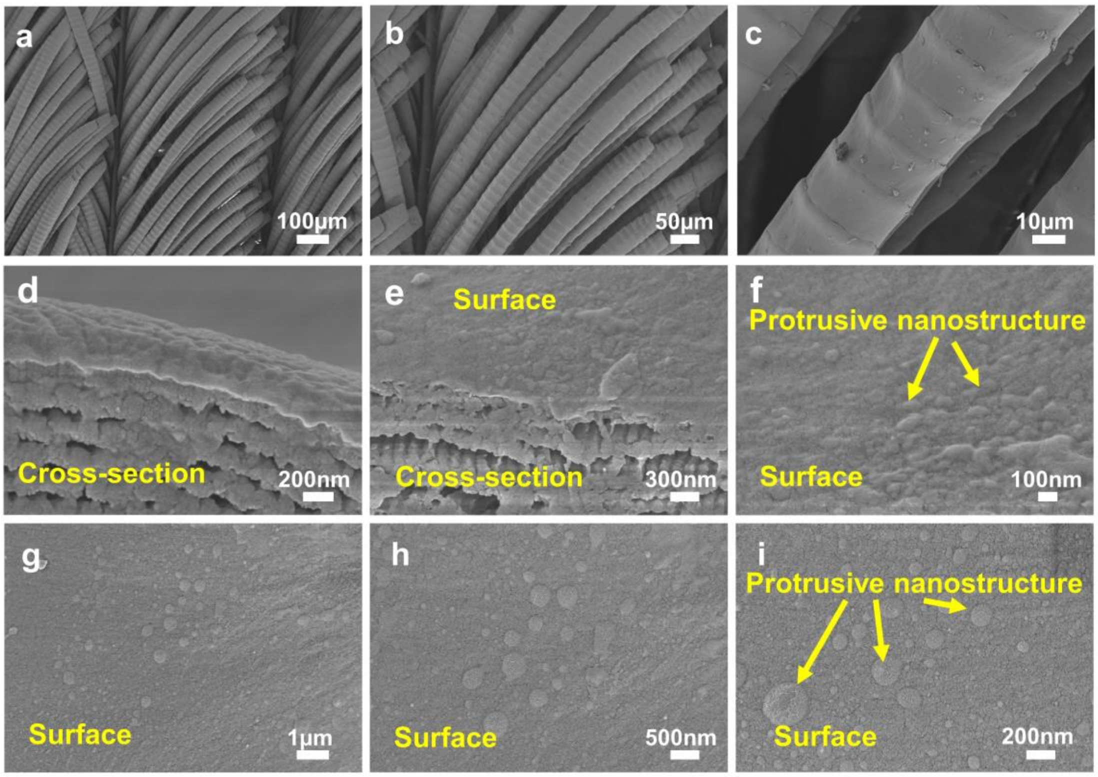

Supplementary Fig. 1 SEM images of peacock back feather. a-c SEM images of the barbule of a peacock back feather. **d** and **e** SEM images of the surface and cross-section structures of the barbule. **f-i** SEM images of surface structures of the barbule.

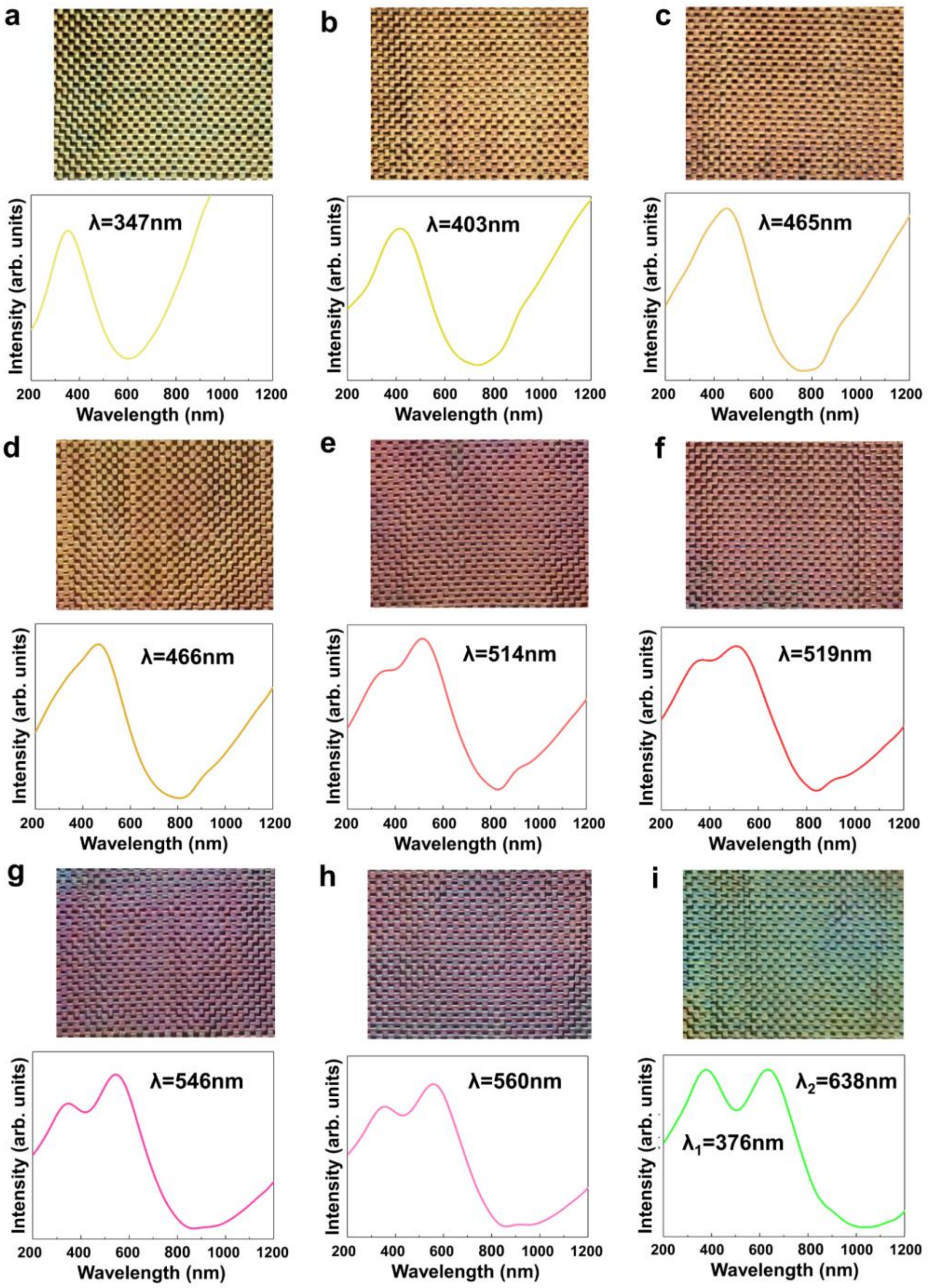

Supplementary Fig. 2 Photographs and the corresponding absorption spectra of various CF fabrics with different glucose solution concentrations. Photographs and the corresponding absorption spectra of **a** 5CC, **b** 6CC, **c** 8CC, **d** 9CC, **e** 11CC, **f** 12CC, **g** 14CC, **h** 15CC, **i** 16CC, respectively.

As shown in Supplementary Fig. 2, photographs of the different colored CF fabrics, derived from glucose reaction concentrations of 5CC, 6CC, 8CC, 9CC, 11CC, 12CC, 14CC, 15CC, and 16CC, were presented by the maximum absorption wavelength of the UV diffuse absorption spectrum to characterize the difference between similar colors.

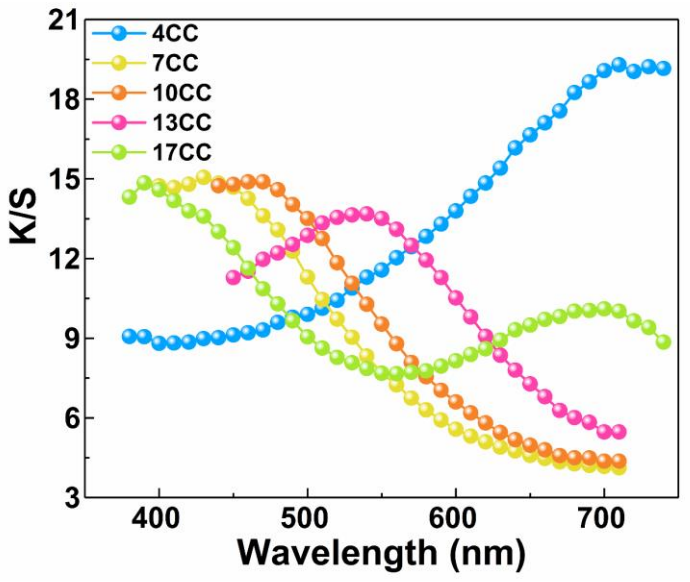

Supplementary Fig. 3 K/S spectra of 4CC, 7CC, 10CC, 13CC, and 17CC.

K/S value is a function (K/S=$(1\text{-}R)^2/2R$) developed by Kubelka and Munk, who theorized in 1931 that the ratio of the coefficient of light absorption (K) to the coefficient of light scattering (S) is corresponding to the fractional reflectance of the light (R) of the opaque substrate at a given wavelength [1, 2]. Here, K/S values of the colored CF fabrics could be directly measured by spectrophotometer at λmax, which was presented with K/S=$(1\text{-}R_{\lambda max})^2/2R_{\lambda max}$. Therefore, the wavelength corresponding to the maximum K/S value (Supplementary Fig. 3), matching with the characteristic wavelengths of reflectance spectra, represented the color strength used to assess the stability of color strength after different treatments.

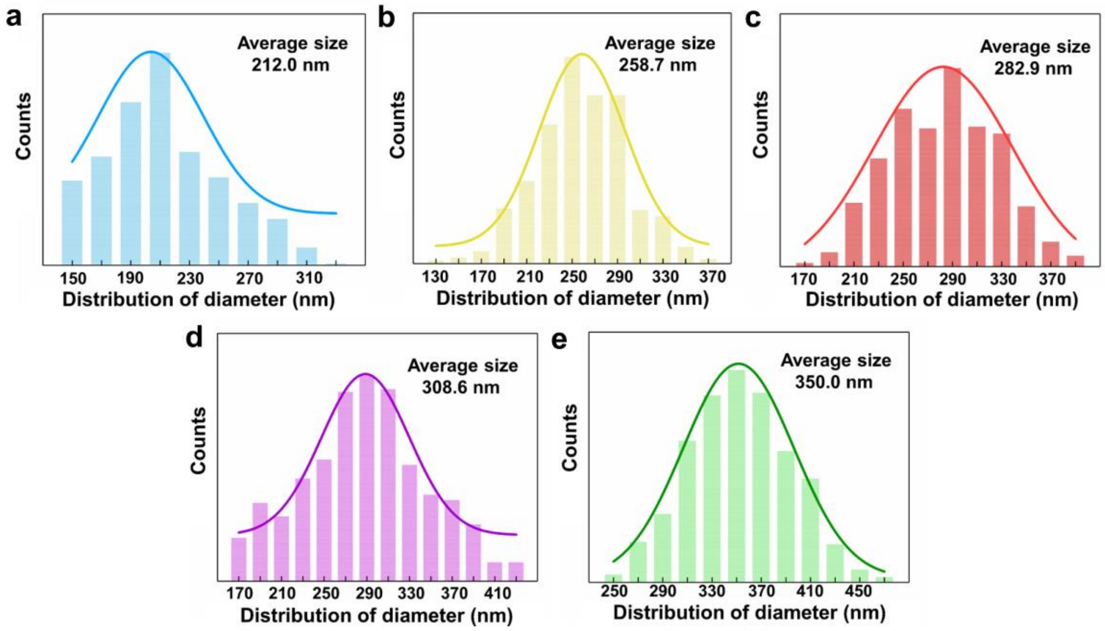

Supplementary Fig. 4 The Gaussian Fitting of CSs nanoparticles diameter distribution. Diameter distribution of **a** 4CC, **b** 7CC, **c** 10CC, **d** 13CC, and **e** 17CC.

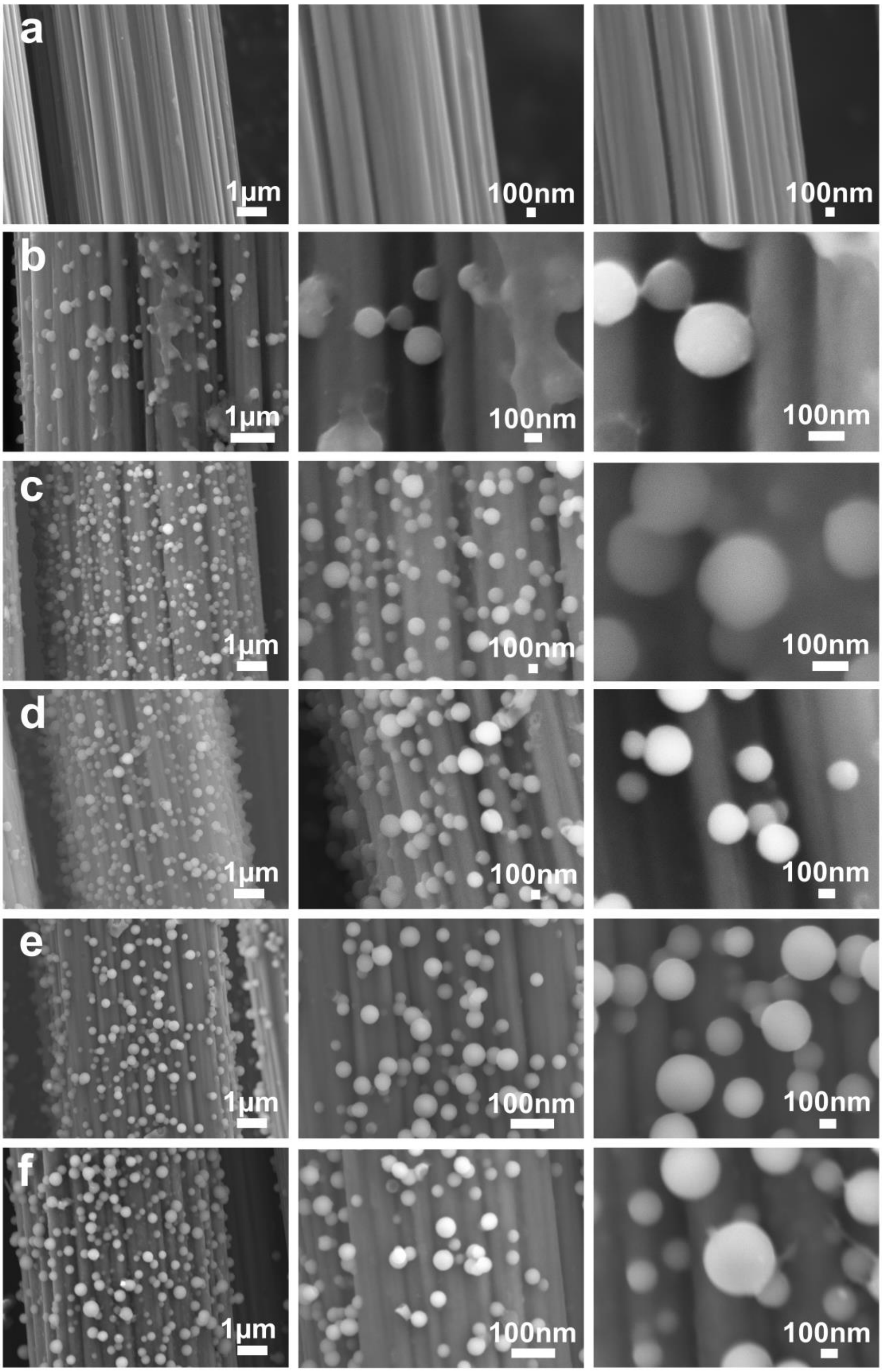

Supplementary Fig. 5 SEM images of as-prepared samples. SEM images of **a** raw CF fabric, **b** 4CC, **c** 7CC, **d** 10CC, **e** 13CC, and **f** 17CC, respectively.

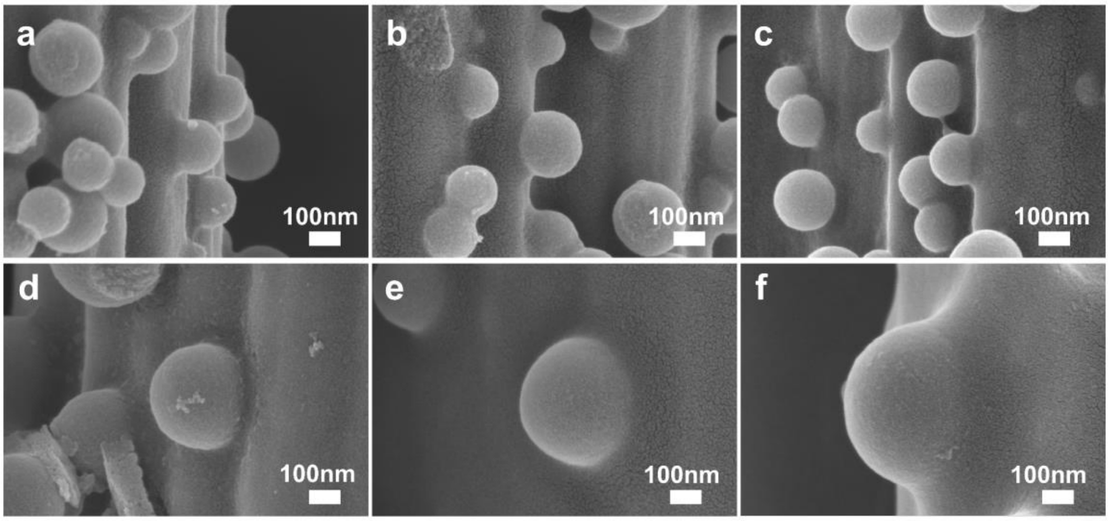

Supplementary Fig. 6 The lateral SEM images of CSs on colored CFs. a-f The high-resolution SEM images of hemispherical contact surface between CSs and CFs at various areas of colored CF fabric.

The high-resolution SEM images of colored CF fabric were carried out to investigate the shape attaching to the surface of CF. It can be seen from Supplementary Figs. 6 a-f, the hemispherical contact surface was indeed displayed from the various areas of colored CF fabric, which contributed to the stability of the structural color.

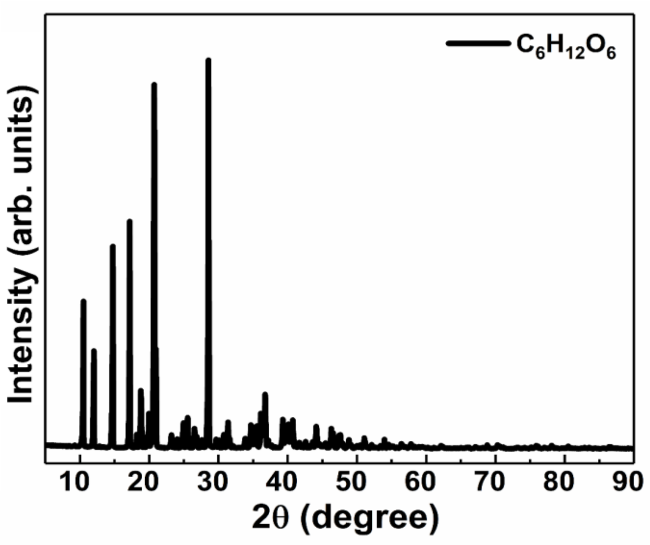

Supplementary Fig. 7 XRD pattern of raw glucose.

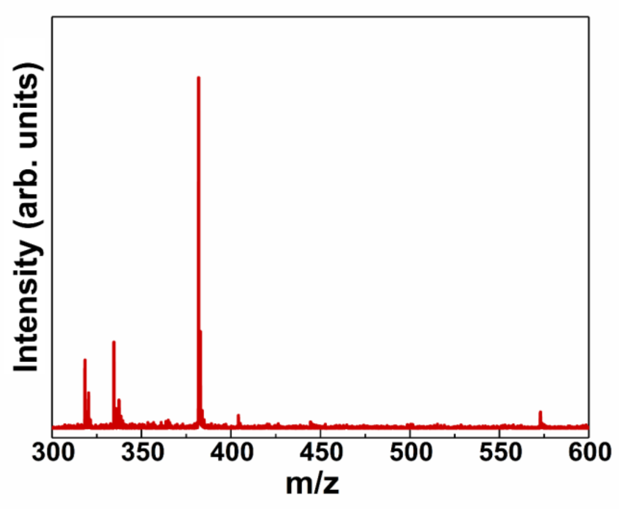

Supplementary Fig. 8 MALDI-TOF mass spectra of solids after the HTC process for 13CC.

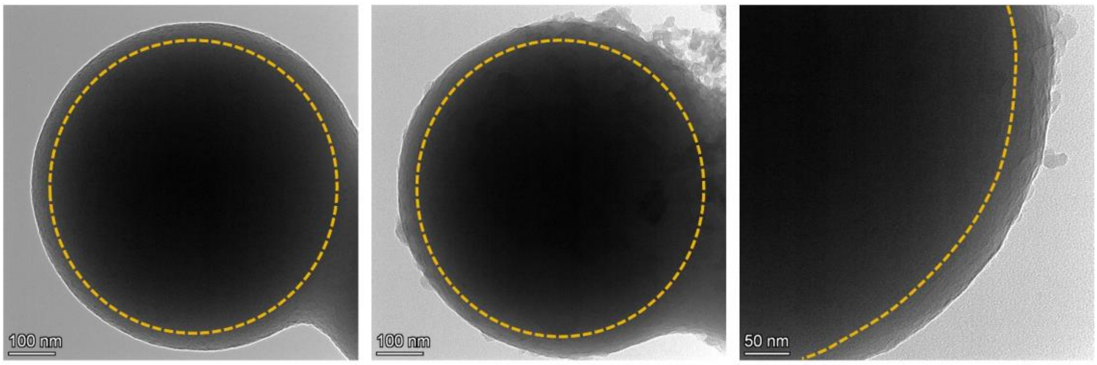

Supplementary Fig. 9 TEM images of CSs for 13CC as the represented sample.

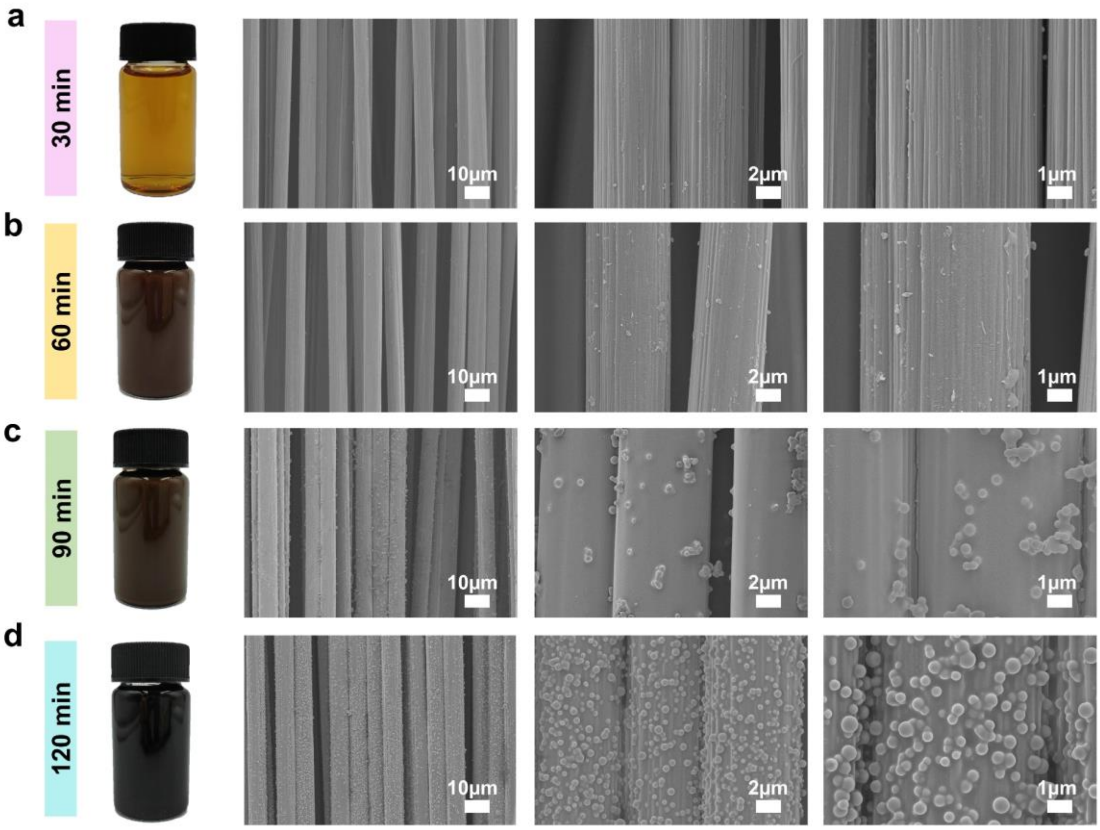

Supplementary Fig. 10 Photographs of the reaction products and SEM images at various reaction times. Photographs of reaction products and SEM images reaction products carbon fibers (CFs) at **a** 30 min, **b** 60 min, **c** 90 min, and **d** 120 min in the HTC reaction process with glucose of 7 g 70 mL^{-1}.

The CSs growth process is investigated by treating the same concentration of glucose with four reaction stages of 30, 60, 90, and 120 min at 250 °C. The colors of reaction liquids were turned from yellow to brownish black with the increase of reaction time (Supplementary Figs. 10 a-d). As shown in Supplementary Fig. 10, SEM images are used to investigate the effects of reaction time on the CSs formation process. At 120 min of hydrothermal reaction (Supplementary Fig. 10 d), a large number of CSs could be observed on the surface of the CFs. These results suggest that there is no aggregation of particles during the initial stage of the reaction, and CSs form gradually on the surface of CF with the time extension in the process of hydrothermal reaction.

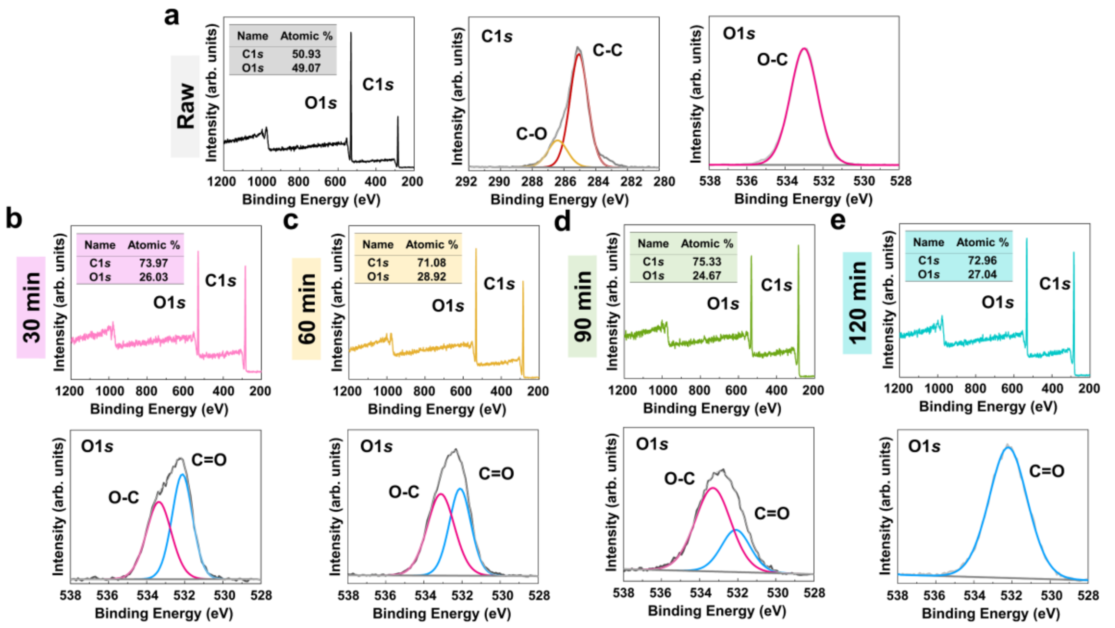

Supplementary Fig. 11 *XPS* survey spectra. a *XPS* survey of C1s, and O1s spectra of glucose. XPS survey spectra and O1s spectra after hydrothermal reaction of glucose at different stages **b** 30 min, **c** 60 min, **d** 90 min, and **e** 120 min at 250 °C.

X-ray photoelectron spectroscopy (*XPS*) was performed to investigate the functional groups of raw glucose and the solid products after hydrothermal reaction with reaction times of 30, 60, 90, and 120 min at 250 °C. As shown in Supplementary Fig. 11, after the hydrothermal reaction, the surface C/O ratio of the solid significantly increased, which is different from the raw glucose (Supplementary Fig. 11 a). Compared to the raw glucose (50.93%), the surface carbon content of solid product increased to 73.97% within 30 min of the reaction. In the subsequent reaction process, the carbon content was maintained in a range of 71.08%–75.33% at different reaction times. In the C1*s* spectra, C–C/C=C, C–O, C=O, and O–C=O are assigned to 285.0, 286.3, 287.5, and 288.9 eV, respectively [3]. The significant changes of the C1*s* *XPS* has been displayed with the forming of C=C, O–C=O, and C=O bonds via the hydrothermal reaction, which is different from the raw glucose (Figs. 2 f-i and Supplementary Fig. 11). The presence of these oxygenated groups was confirmed by the O1*s* spectrum. The signals of O1*s* spectra attribute to the O-C (531.0 eV), and O=C (532.1 eV) [4], which confirm the formation of oxygenated groups O=C bonds during the hydrothermal reaction [5]. The oxygen-containing functional groups present on the surface of solid products may consist of more reactive/ hydrophilic groups [6]. Combining with the photographs of

products (Supplementary Fig. 10) and SEM images at different reaction stages, Supplementary Fig. 11 shows the changes of functional group for the products at the various reaction stages.

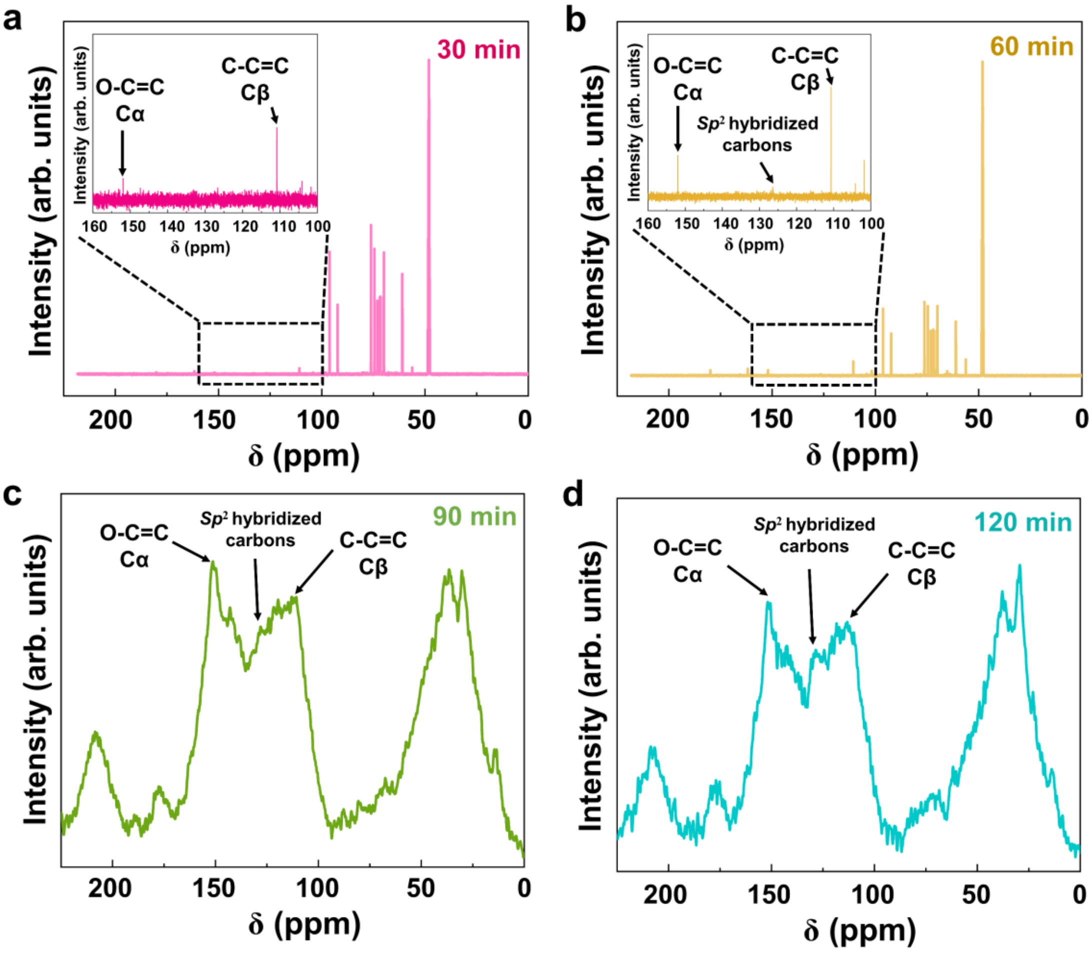

Supplementary Fig. 12 NMR spectra of as-prepared samples at 250 °C with the extension of time. Solution ^{13}C NMR spectra of **a** 30 min and **b** 60 min after hydrothermal reaction of glucose at 250 °C. Solid ^{13}C NMR spectra of **c** 90 min and **d** 120 min after hydrothermal reaction of glucose at 250 °C.

Supplementary Fig. 12 shows the 110–150 ppm region during the hydrothermal reaction of glucose at 250 °C with the extension of time. In the early stages of the reaction, the region is characterized by the presence of two peaks, which are due to the furanic rings (140–153 and 110–120 ppm). At 30 min, the products obtained from glucose of hydrothermal have a polymer-like structure composed of polyfuranic chains domains [7], along with the practical absence of a central peak at 125–129 ppm (Supplementary Fig. 12 a). As the HTC residence time increases, the relative intensity of the central peak at 125–129 ppm starts forming (Supplementary Fig. 12 b) with the production of solids (Supplementary Fig. 10 c). Corresponding to the SEM images, the NMR spectra show the relative intensity of the peaks at 140−153 ppm, and 110−120 ppm are assigned to the carbons of Cα and Cβ assigned to the furanic ring. With the

extension of reaction time, the obvious intensity of the peak at 125–129 ppm is observed, which can be assigned to carbon atoms belonging to aromatic rings, demonstrating the aromatization of the polyfuranic compounds. Compared to the solid ^{13}C NMR spectra of 90 min, the relative intensity of the peak at 125–129 ppm enhanced in Supplementary Fig. 12 d, demonstrating the increased degree of aromatization. As shown in Supplementary Figs. 10-12, due to the intramolecular dehydration, condensation, and decarboxylation, the more condensed sp^2 hybridized-aromatic chemical species was created [8]. Nucleation then occurs at the critical supersaturation point of insoluble clusters. Furthermore, the active groups of the surface promote the growth and formation of CSs with the enhancement of hydrothermal carbonization [6, 9].

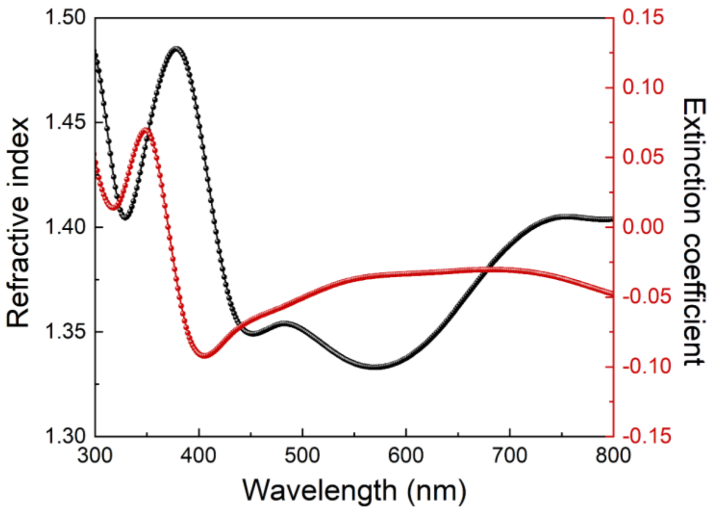

Supplementary Fig. 13 Complex refractive index of CSs (Complex refractive index $\tilde{\boldsymbol{n}} = n_r + ki$).

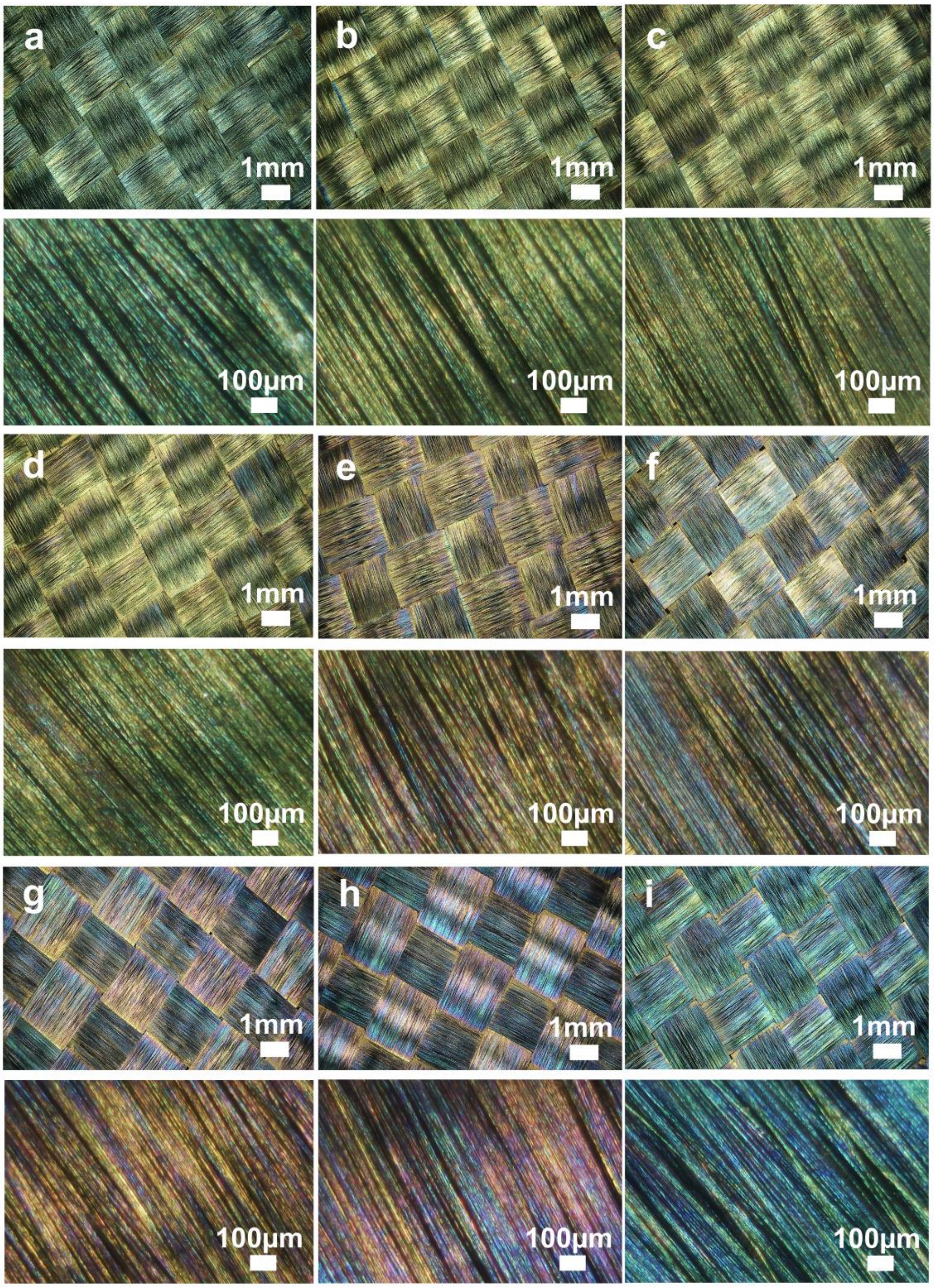

Supplementary Fig. 14 3D optical microscope images of CF fabrics for various glucose solution concentrations. 3D optical microscope images of **a** 5CC, **b** 6CC, **c** 8CC, **d** 9CC, **e** 11CC, **f** 12CC, **g** 14CC, **h** 15CC, **i** 16CC, respectively.

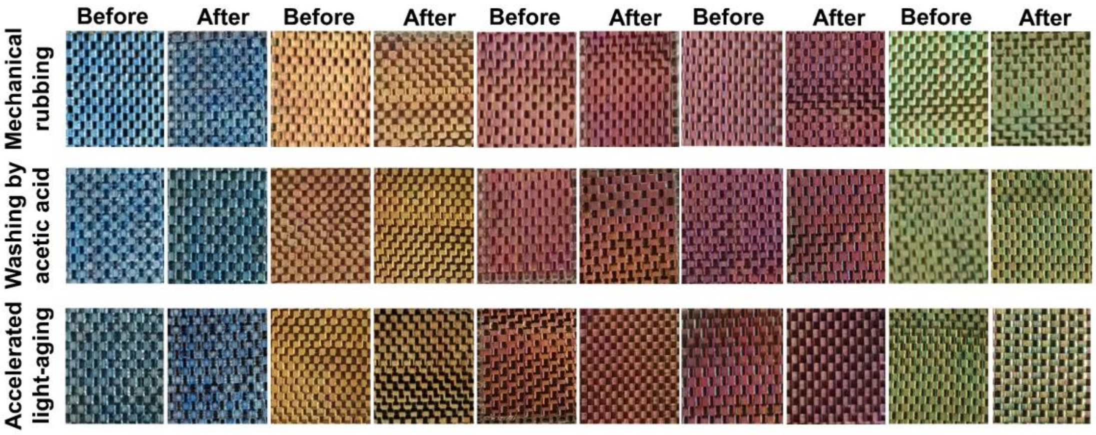

Supplementary Fig. 15 Photographs of the fabricated CF fabrics before and after mechanical rubbing test performed by color fastness friction meter with a load pressure of 50 kPa for 10 cycles, the soaking and washing of acetic acid solution test after mechanical rubbing test at a vibration of 60 times per minute for 120 min, the accelerated light-aging test under the strong light using a xenon lamp light source system for simulated sunlight after mechanical rubbing test and soaking and washing of acetic acid solution, with the environment temperature, humidity, and light irradiance were set as 38 °C, 47 % RH, and 1.271×10^4 W m^{-2}, for 4CC, 7CC, 10CC, 13CC, and 17CC, respectively.

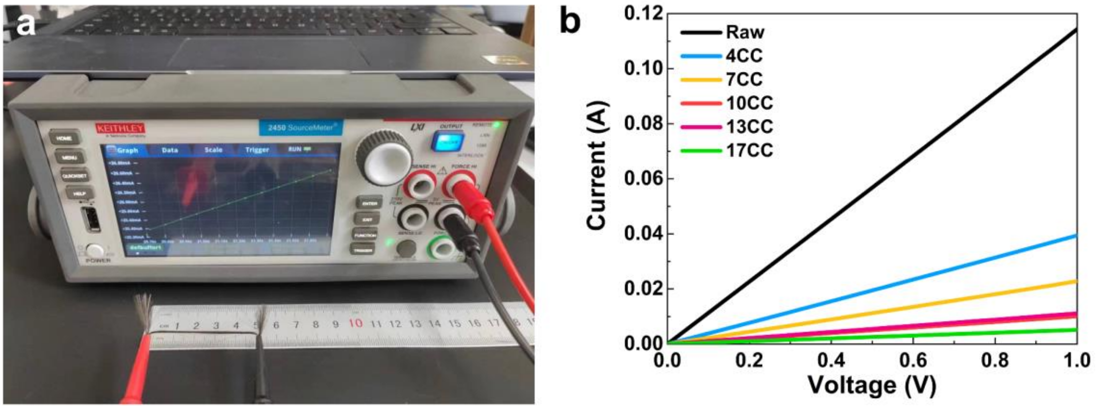

Supplementary Fig. 16 Electrical performance test of as-prepared samples. a Operating process of electrical performance test. **b** Current-voltage curves of fibers bundle driven from the raw carbon fiber fabric and colored fiber fabric.

The bundle of CFs is clamped to the electrodes with test length of 5 cm, and the range of test voltage is set at 0.0 -1.0 V. The operating process is shown in Supplementary Fig. 16 a. Herein, the bundle of CFs was derived from the raw and colored CF fabric. Supplementary Fig. 16 b showed that the slope of current-voltage curves was reduced for the colored CF, which demonstrated that the electrical conductivity of raw CFs was better than that of colored CFs. This may be mainly attributed to the increase in the electrical contact resistance of the colored CF surface. Additionally, the stable surface contact between CSs and CFs may also contribute to this phenomenon.

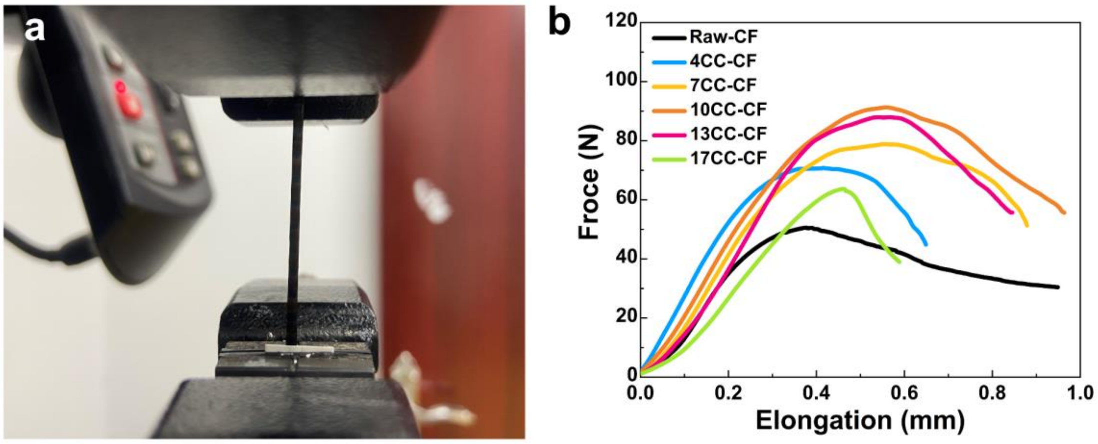

Supplementary Fig. 17 Mechanical properties of as-prepared samples. a Operating process of mechanical performance test. **b** Mechanical properties of carbon fibers bundle driven from the raw carbon fiber fabric and colored fiber fabric.

As demonstrated in Supplementary Fig. 17 a, the mechanical performance of the CFs bundle, which was driven from the raw or colored CF fabric, was tested by the Instron. The CSs coatings exhibited an enhancement influence on the mechanical performance of colored CFs. As shown in Supplementary Fig. 17 b, compared with pristine CFs, the tensile strength of colored CFs was enhanced.

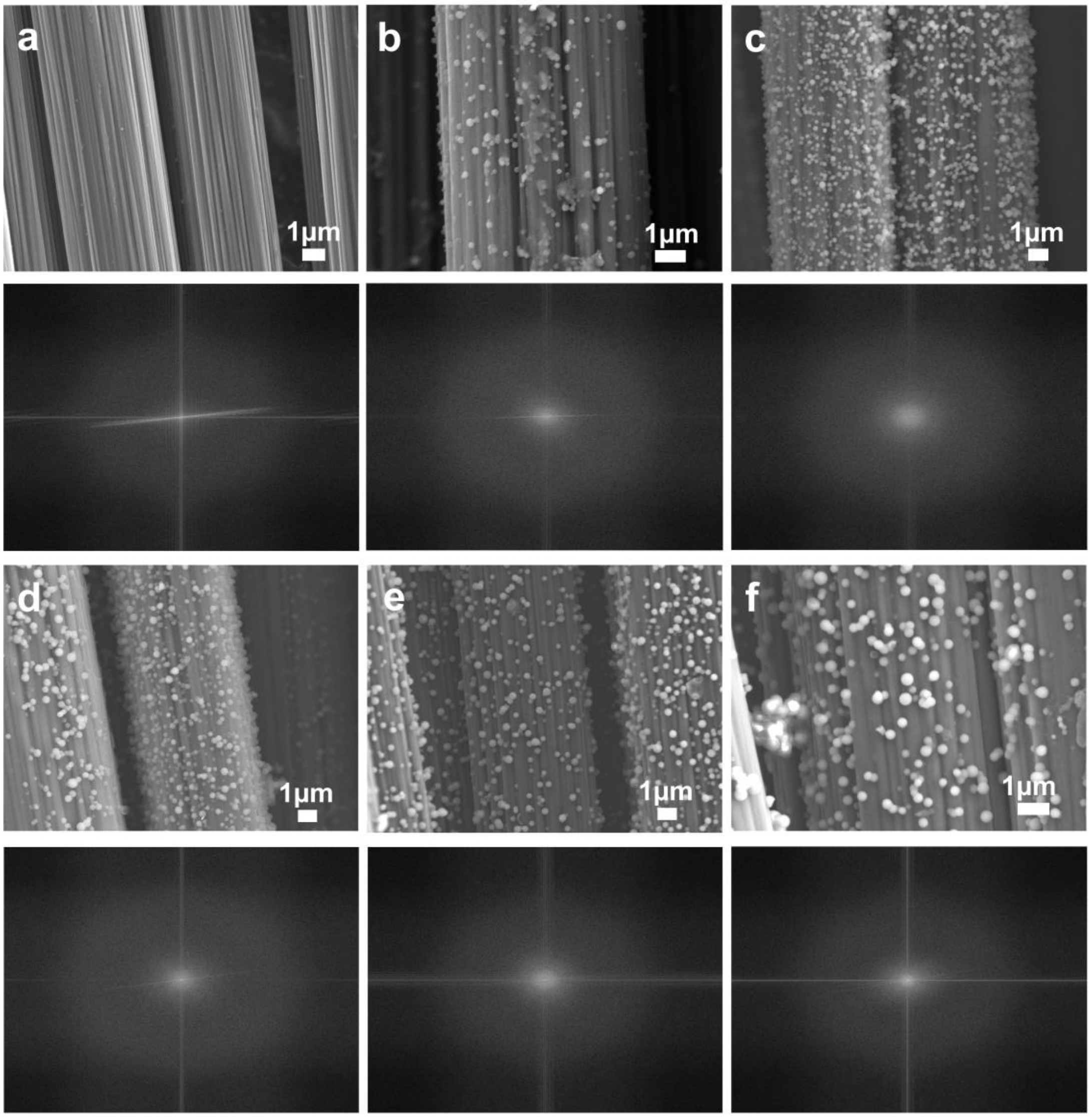

Supplementary Fig. 18 SEM images and the 2D Fourier transform (FT) of the corresponding arrays to SEM images. SEM images and the 2D FT of **a** raw CF fabric, **b** 4CC, **c** 7CC, **d** 10CC, **e** 13CC, and **f** 17CC, respectively.

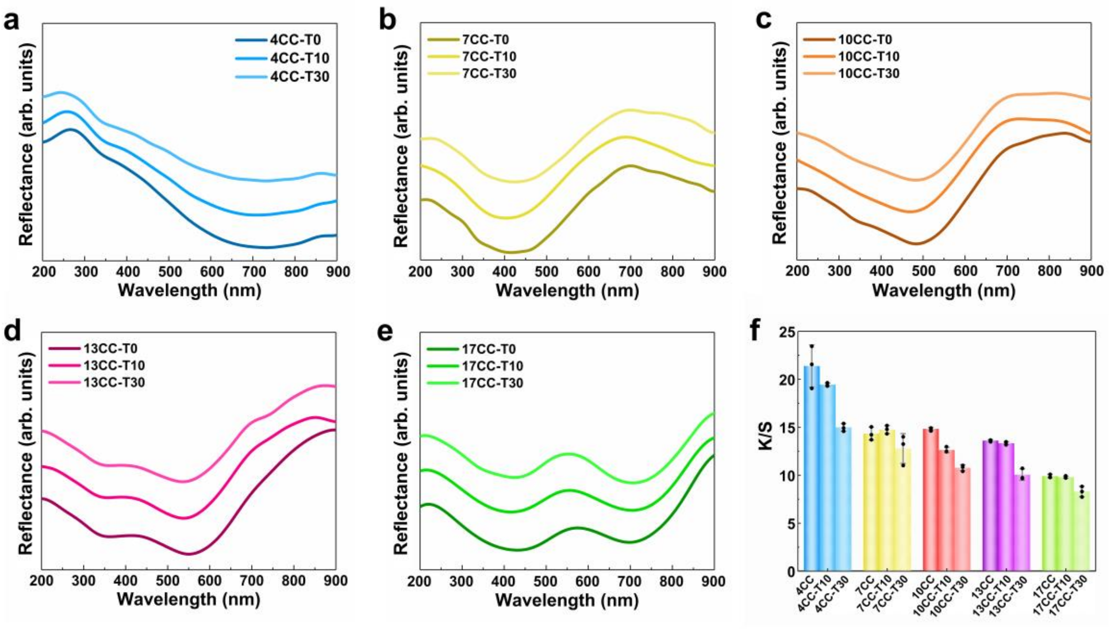

Supplementary Fig. 19 Color stability after mechanical rubbing test of cycling 0, 10, 30 times. **a-e** The measured reflectance spectra of colored CF fabric for 4CC, 7CC, 10CC, 13CC, and 17CC. **f** K/S values of these samples. Error bars represent standard deviation based on the different positions (n=3). Tx; T: mechanical rubbing test; x: the cycle times.

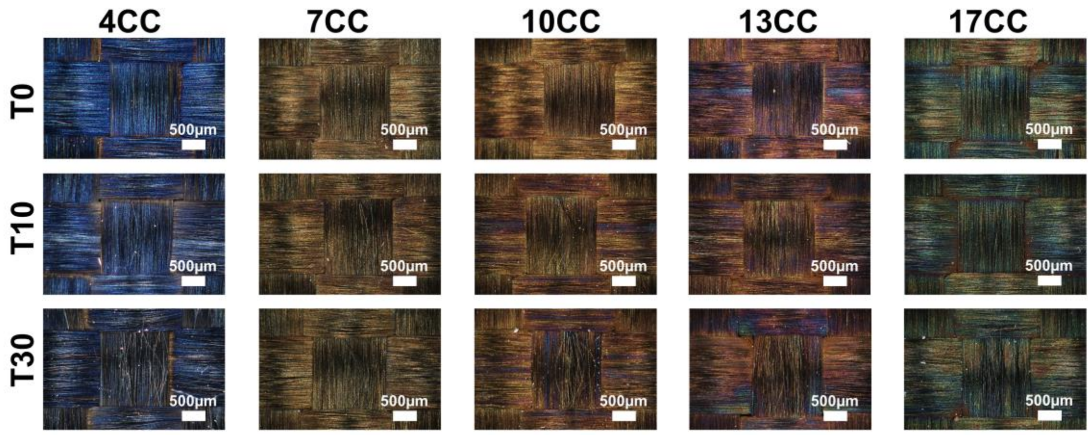

Supplementary Fig. 20 3D optical microscope images after mechanical rubbing test of cycling 0 (T0), 10 (T10), 30 (T30) times for 4CC, 7CC, 10CC, 13CC, and 17CC. Tx; T: mechanical rubbing test; x: the cycle times.

Supplementary Table 1 Carbon content of hydrochars derived by glucose.

Feedstock	Sample	Temperature (°C)	Time (h)	Carbon (%)
Glucose	13CC	250	2	67.17

The reaction temperature offers two fundamental functions, providing heat for dissociation to accelerate the polymerization and aromatization reactions [10]. Meanwhile, the residence time determines the extent of carbonization at a given temperature [11]. The evolution of the carbon-related chemical structures in the HTC process was affected by the combined influence of temperature and residence time, which maintained constant during the reactions. Supplementary Table 1 shows that the value of carbon content was 67.17 % under the reaction temperature of 250 °C and time of 2 h, thus producing a carbon-rich CSs [12, 13].

Supplementary Table 2 Different methods of preparing structurally colored CFs.

Substrate	Pretreatment	Materials	Synthetic method	Forming method	Structure	Control method	Color Category	Ref.
CF	Nitric acid	Al_2O_3/TiO_2		Magnetron sputtering	1D Photonic crystal	Sputtered thickness	Four	[14]
CF		TiO_2		Atomic layer deposition	Dense coating	Coating thickness	Four	[15]
CF	Plasma	Poly(St-MMA-AA)	Emulsion polymerization	Gravity Sedimentation	Photonic crystal	Particle sizes	Five	[16]
CF	Plasma	$Al(CH_3)_3$ / $Zn(CH_2CH_3)_2$	Hydrolysis	Atomic layer deposition	Photonic crystal	Layer thickness	Five	[17]
CF		PS	Emulsion polymerization	Electrophoretic Deposition	Photonic crystal	Particle sizes	Three	[18]
CF		Acrylic acid /aryldiazonium salts	Polymerization grafting	Electro initiated emulsion polymerization	Transparent thin film	Polymer swells by acetone	Four	[19]
CF		FeOOH		Hydrothermal method	Uniform nanoparticle	Particle sizes	Four	[20]
CF		PMMA	Free radical polymerization	Electrophoretic deposition	Photonic crystal	Particle sizes	Four	[21]
CF		PNIPAM-co-AAc diblock copolymers	Emulsion polymerization	Electrophoretic deposition	Photonic crystal	Particle sizes	Three	[22]
CF		Glucose		Hydrothermal reaction	Disordered CS particles	Mean particle sizes	Five	This work

Supplementary Table 3 The K/S value at three different positions of colored CF fabric (R), after mechanical rubbing test (T), acid pickling (A), and accelerated light-aging test (L) for colored CF fabric, successively.

Sample	Characteristic Wavelength	Different process	K/S value at different positions 1	2	3	Mean	Standard
4CC	700	R	19.30	21.81	23.58	21.56	2.15
		T	19.29	19.40	19.62	19.44	0.17
		A	19.26	18.30	19.55	19.04	0.65
		L	16.87	16.68	16.38	16.64	0.25
7CC	430	R	13.72	14.24	15.06	14.34	0.67
		T	14.31	14.77	15.16	14.75	0.42
		A	13.51	14.10	14.84	14.15	0.67
		L	14.07	13.87	14.12	14.02	0.13
10CC	470	R	14.53	14.67	15.35	14.85	0.44
		T	13.68	13.10	14.40	13.73	0.65
		A	13.43	14.35	14.26	14.01	0.51
		L	14.40	13.56	13.11	13.69	0.66
13CC	540	R	13.35	13.42	13.88	13.55	0.29
		T	12.18	13.10	12.42	12.57	0.48
		A	13.18	12.98	12.79	12.98	0.20
		L	13.43	13.68	13.40	13.50	0.15
17CC	700	R	9.78	9.83	10.11	9.91	0.18
		T	9.70	9.76	9.91	9.79	0.11
		A	9.64	9.74	9.74	9.70	0.06
		L	10.26	10.26	9.57	10.03	0.40

Supplementary Table 4 Average K/S values after mechanical rubbing. Tx; T: mechanical rubbing test; x: the cycle times.

Sample	Mechanical rubbing	K/S value at different positions			Mean	Standard
		1	2	3		
4CC	T0	19.08	21.58	23.47	21.38	2.20
	T10	19.29	19.40	19.62	19.44	0.17
	T30	14.61	15.40	14.94	14.98	0.40
7CC	T0	14.24	15.06	13.72	14.34	0.67
	T10	14.31	14.77	15.16	14.75	0.42
	T30	11.03	13.27	14.02	12.78	1.56
10CC	T0	14.94	14.64	14.89	14.82	0.16
	T10	12.49	12.44	12.96	12.63	0.29
	T30	10.43	11.03	10.87	10.77	0.31
13CC	T0	13.51	13.64	13.68	13.61	0.09
	T10	13.46	13.36	13.16	13.33	0.16
	T30	10.73	9.75	9.68	10.06	0.59
17CC	T0	9.79	9.83	10.11	9.91	0.17
	T10	8.84	8.32	7.75	8.30	0.55
	T30	9.70	9.76	9.91	9.79	0.11

Supplementary Note 1. Analysis of CSs FTIR spectra for various glucose concentrations

Fig. 2c presents recognizable peaks of the CSs FTIR spectra for various glucose concentrations. The characteristic peak of C−O deformation in the furan rings at 1020 cm^{-1} confirms the structural motif of the furan ring. The two peaks at 1700 and 1610 cm^{-1} were assigned to C=O stretching and C=C stretching, respectively, indicating the presence of aromatic units and carbonyl groups in saturated aliphatic ketones [23]. The relative intensity at 1510 cm^{-1} was ascribed to furan ring stretching, and the obvious peaks ranging from 710 to 820 cm^{-1} were interpreted as C−H for the out-of-plane deformation of furan [24].

Supplementary Note 2. Solid-state carbon-13 MAS NMR spectra

As displayed in Fig. 2d, the peak assignments could be divided into three main regions. The signals within $\delta < 90$ ppm were attributed to primary, secondary, and tertiary aliphatic C atoms [25]. The characteristic features in the $\delta > 150$ ppm portion corresponded to ketones, carboxylic, and ester functional groups. Three consecutive signals at $\delta < 50$ ppm reflected the presence of methyl groups, $-CH_2$, and C−H/C, respectively [24].

Supplementary References

1. Kale, B.M. et al. Dyeing and stiffness characteristics of cellulose-coated cotton fabric. *Cellulose* **23**, 981-992 (2016).
2. Becerir, B. A novel approach for estimating the relation between K/S value and dye uptake in reactive dyeing of cotton fabrics. *Fibers Polym.* **6**, 224-228 (2005).
3. Yu, S. et al. Decoupled temperature and pressure hydrothermal synthesis of carbon sub-micron spheres from cellulose. *Nat. Commun.* **13**, 3616 (2022).
4. Wang, F. et al. Selective adsorption–deposition of gold nanoparticles onto monodispersed hydrothermal carbon spherules: a reduction–deposition coupled mechanism. *J. Mater. Chem. A* **3**, 1666 (2015).
5. Ryu, J., Suh, Y.W., Suh, D.J. & Ahn, D.J. Hydrothermal preparation of carbon microspheres from mono-saccharides and phenolic compounds. *Carbon* **48**, 1990-1998 (2010).
6. Sevilla, M. & Fuertes, A.B. Chemical and structural properties of carbonaceous products obtained by hydrothermal carbonization of saccharides. *Chem. Eur. J.* **15**, 4195-4203 (2009).
7. Falco, C., Baccile, N. & Titirici, M.M. Morphological and structural differences between glucose, cellulose and lignocellulosic biomass derived hydrothermal carbons. *Green Chem.* **13**, 3273 (2011).
8. Baccile, N. et al. Structural characterization of hydrothermal carbon spheres by advanced solid-state MAS ^{13}C NMR Investigations. *J. Phys. Chem. C* **113**, 9644–9654 (2009).
9. Sevilla, M. & Fuertes, A.B. The production of carbon materials by hydrothermal carbonization of cellulose. *Carbon* **47**, 2281-2289 (2009).
10. Nizamuddin, S. et al. An overview of effect of process parameters on hydrothermal carbonization of biomass. *Renew. Sust. Energ. Rev.* **73**, 1289-1299 (2017).
11. Funke, A. & Ziegler, F. Hydrothermal carbonization of biomass: A summary and discussion of chemical mechanisms for process engineering. *Biofuel. Bioprod. Biorefin.* **4**, 160-177 (2010).
12. Sevilla, M., Fuertes, A.B. & Mokaya, R. High density hydrogen storage in superactivated carbons from hydrothermally carbonized renewable organic materials. *Energy Environ. Sci.* **4**, 1400 (2011).
13. Jung, D., Zimmermann, M. & Kruse, A. Hydrothermal carbonization of fructose: growth mechanism and kinetic model. *ACS Sustain. Chem. Eng.* **6**, 13877-13887 (2018).
14. Zhao, K. et al. Photonic Janus carbon fibers with structural color gradient for multicolored, wirelessly wearable thermal management devices. *Adv. Mater. Technol.* **7**, 2101057 (2021).
15. Chen, F. et al. Facile and effective coloration of dye-inert carbon fiber fabrics with tunable colors and excellent laundering durability. *ACS Nano* **11**, 10330-10336 (2017).
16. Yu, J., Lee, C.H., Kan, C.W. & Jin, S. Fabrication of structural-coloured carbon fabrics by thermal assisted gravity sedimentation method. *Nanomaterials* **10**, 1133 (2020).
17. Niu, W. et al. Multicolored photonic crystal carbon fiber yarns and fabrics with mechanical robustness for thermal management. *ACS Appl. Mater. Inter.* **11**, 32261-32268 (2019).
18. Zhou, N., Zhang, A., Shi, L. & Zhang, K.Q. Fabrication of structurally-colored fibers with axial core-shell structure via electrophoretic deposition and their optical properties. *ACS Macro Lett.* **2**, 116-120 (2013).
19. Eyckens, D.J. et al. Fiber with butterfly wings: creating colored carbon fibers with

increased strength, adhesion, and reversible malleability. *ACS Appl. Mater. Inter.* **11**, 41617-41625 (2019).

20. Lin, Z. et al. High structural stability of colored carbon fiber cloths modified by FeOOH. *Appl. Surf. Sci.* **545**, 148994 (2021).
21. Liu, Z., Zhang, Q., Wang, H. & Li, Y. Structurally colored carbon fibers with controlled optical properties prepared by a fast and continuous electrophoretic deposition method. *Nanoscale* **5**, 6917-6922 (2013).
22. Yuan, X. et al. Visibly vapor-responsive structurally colored carbon fibers prepared by an electrophoretic deposition method. *RSC Adv.* **6**, 16319-16322 (2016).
23. Modugno, P. & Titirici, M.M. Influence of reaction conditions on hydrothermal carbonization of fructose. *ChemSusChem* **14**, 5271-5282 (2021).
24. Ilona, V.Z. et al. Formation, molecular structure, and morphology of humins in biomass conversion: influence of feedstock and processing conditions. *ChemSusChem* **6**, 1745-1758 (2013).
25. Ilona, V.Z. et al. Structural characterization of 13C-enriched humins and alkali-treated 13C humins by 2D solid-state NMR. *Green Chem.* **17**, 4383-4392 (2015).
