# Supplementary figures and images for: Color construction of multi-colored carbon fibers using glucose
Source: Nat Commun. 2024 Mar 4;15:1979. doi: 10.1038/s41467-024-46395-5 (PMC10912437; doi:10.1038/s41467-024-46395-5)

## Slide 1
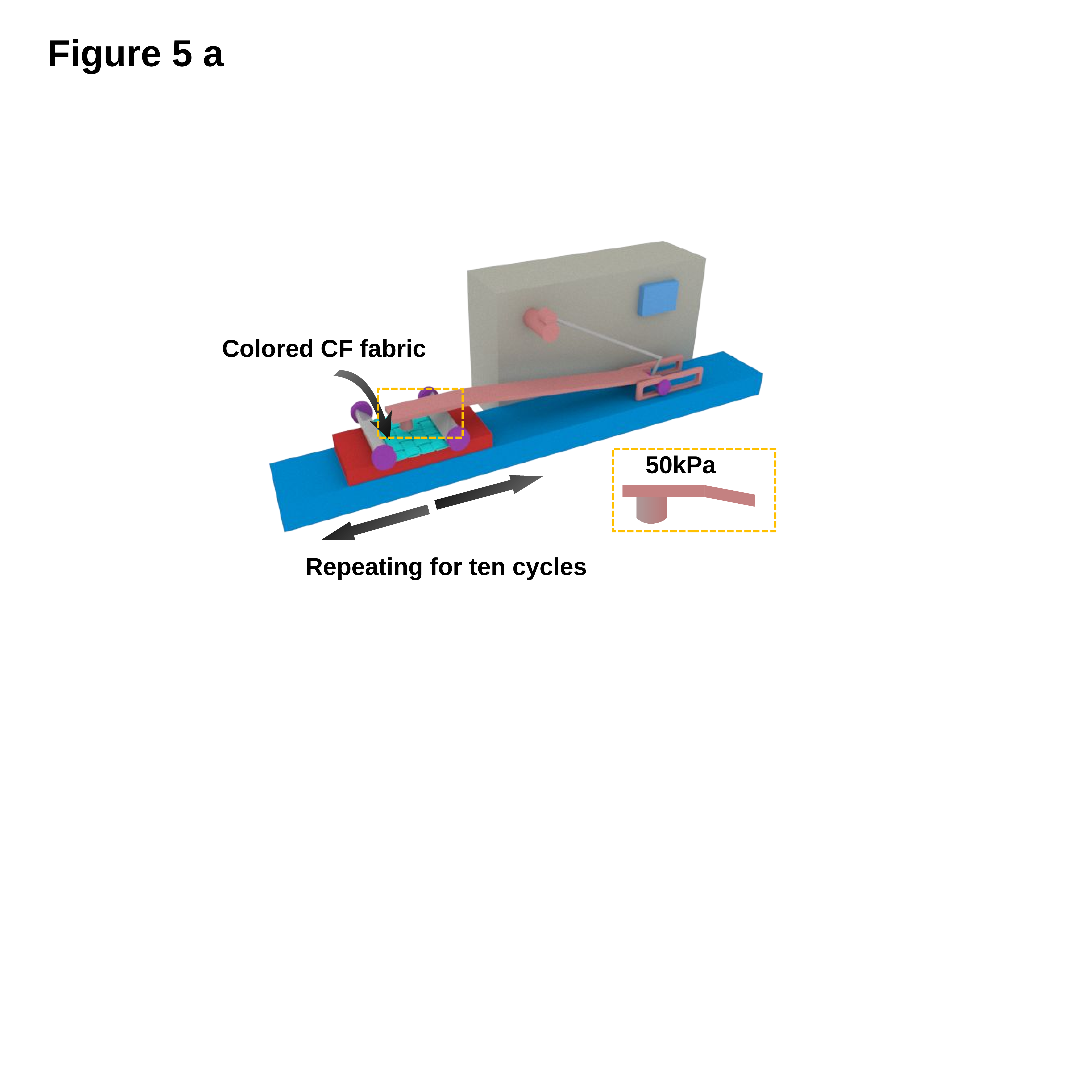

Figure 5 a
Colored CF fabric
50kPa
Repeating for ten cycles

## Slide 2
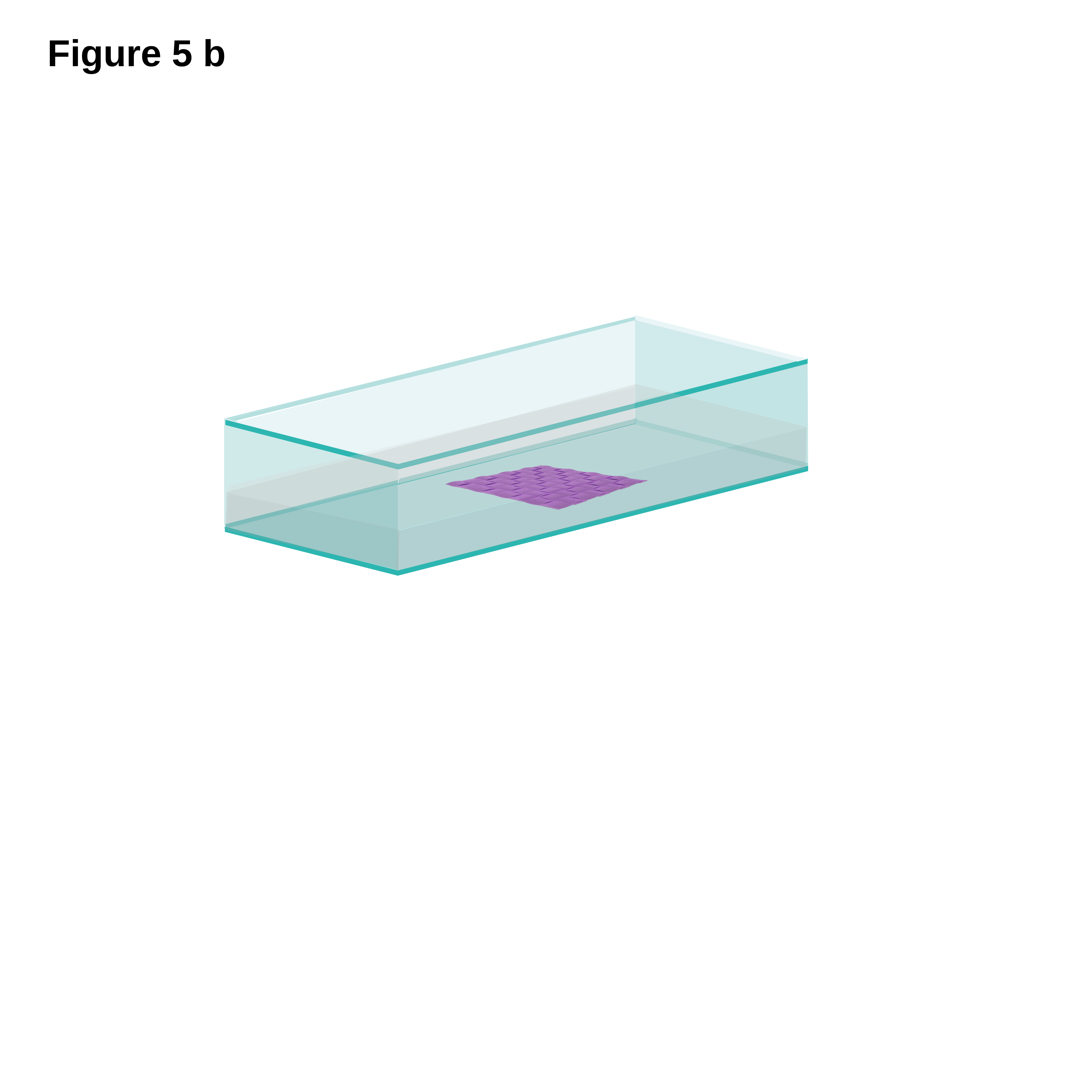

Figure 5 b

## Slide 3
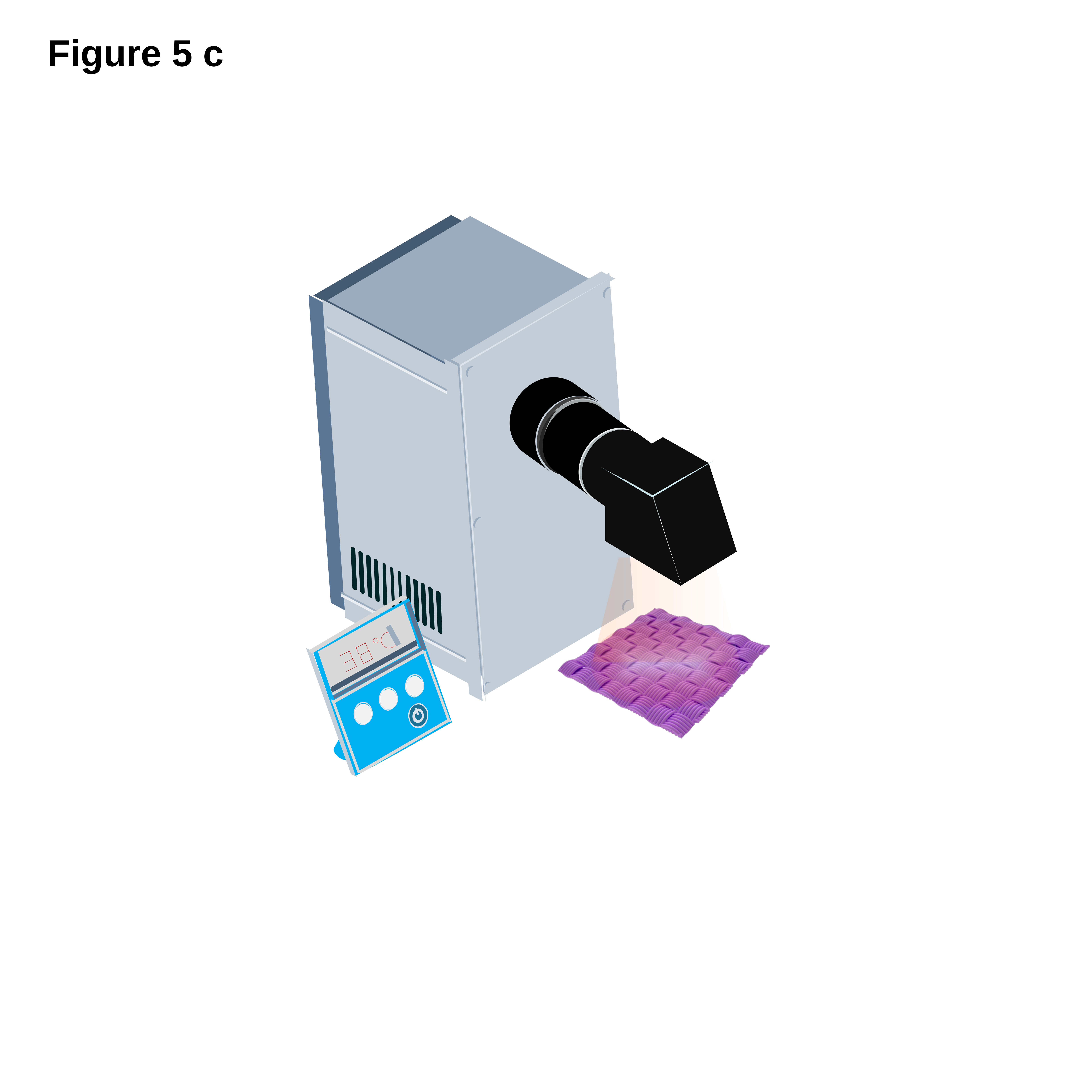

Figure 5 c

Supplement: Supplementary file 3 — Source data [file 41467_2024_46395_MOESM3_ESM.zip › SourceData/Images source file of Figure 5a-c/Source file Figure 5 a-c.pptx]
